# A retrospective case series and technological review of post-surgical analgesia for orthopedic surgery using a smart connected infusion pump: an opioid-sparing protocol

**DOI:** 10.3389/fphar.2026.1838931

**Published:** 2026-10-27

**Authors:** David Nelson, Jeffrey Brand, Virginia Rubio, Anna Lester

**Affiliations:** 1 Georgetown Surgery Center, Georgetown, TX, United States; 2 Eitan Medical, Netanya, Israel

**Keywords:** infusion pump, opioid-sparing analgesia, perineural infusion, regional anesthesia, smart pump

## Abstract

**Background:**

Opioid-sparing analgesia (OSA) strategies aim to reduce perioperative opioid exposure while maintaining effective pain control. Continuous peripheral nerve blocks (cPNBs) supported by ambulatory infusion pumps have demonstrated efficacy in reducing opioid consumption, yet data on electronic infusion systems in the home environment remain limited.

**Methods:**

A retrospective, single-center, uncontrolled feasibility series evaluated the performance of a smart connected electronic infusion pump within a multimodal opioid-sparing postoperative analgesia protocol in routine clinical practice. Twenty orthopedic surgery patients with continuous peripheral nerve catheters and patient-controlled boluses received an initial 20 mL nerve block (adductor canal, with or without supplemental iPACK, or interscalene/infraclavicular brachial plexus) followed by 0.2% ropivacaine via a standardized infusion regimen (8 mL/h basal rate with 3 mL patient-controlled bolus, 30-min lockout).

**Results:**

Postoperative opioid consumption over 3 days varied by procedure type and regional anesthesia technique. Overall, a median of 15 MME (IQR: 0–39.4 MME, range: 0–90 MME). Shoulder surgery patients (n = 12, 60% of cohort) receiving interscalene brachial plexus catheters had a median consumption of 15 MME (IQR: 0–45 MME, range: 0–90 MME), with 42% (5/12) using no opioids. ACL reconstruction patients (n = 3, 15% of cohort) receiving adductor canal blocks had even lower consumption, with a median of 0 MME (range: 0–22.5 MME) and 67% (2/3) reporting no opioid use. Patients undergoing other procedures (TKA, PFL and humerus fracture; n = 4, 20% of cohort) had a median consumption of 41.25 MME (range: 7.5–52.5 MME), and the thumb surgery patient (5%) reported no opioid use. Seventeen patients (85%) completed the full programmed infusion; the remaining three (15%) discontinued early due to catheter dislodgement, not pump malfunction. All alarms were resolved at home without interrupting therapy, and no pump-related discontinuations occurred.

**Conclusion:**

In this small, real-world retrospective, single-center, uncontrolled feasibility series, a smart electronic infusion pump reliably delivered opioid-sparing perineural analgesia within a multimodal OSA protocol following orthopedic surgery in ambulatory and home settings, with no pump-related discontinuations and successful patient management of alarms. These observations support feasibility; analgesic adequacy was assessed only as a binary outcome (pain controlled or uncontrolled). Future comparative studies evaluating different ambulatory infusion platforms would help identify optimal device characteristics for home-based perineural analgesia.

## Introduction

In recent years there has been increasing interest in Opioid-Sparing Analgesia (OSA) approaches to pain management, considering the risks of increased hospital morbidity (Santosa et al., 2023) and development of postoperative Persistent Opioid Use. Post-surgical opioids have increased risk if patients are i) opioid naive ii) undergoing orthopedic surgery iii) larger initial opioid prescriptions, and iv) longer duration of initial prescriptions (Gong et al., 2024; Gil et al., 2019; Gossett et al., 2019). OSA emerged as a strategy to enhance the therapeutic benefit-to-harm ratio by limiting perioperative opioid exposure to achieve analgesia during and after surgery, replacing traditional opioid-based anesthesia (Lauretta et al., 2025). The traditional practice of prescribing 20–80 opioid tablets (historically >400 MME) after shoulder surgery significantly exceeds actual patient needs when multimodal analgesia and regional blocks are used (Welton et al., 2018).

Multimodal opioid-sparing protocols have become the standard of care in perioperative pain management for shoulder or knee surgery (The NO Pain Investigators et al., 2022), but without studies assessing real world protocols and implementation with infusion pumps. A prospective study of 100 rotator cuff repair patients with interscalene blocks found that 77% required fewer than 15 tablets of oxycodone 5 mg (112.5 MME), with high patient satisfaction (Mandava et al., 2021). While opioid-sparing strategies have shown high patient satisfaction across many surgical fields, documented risk of persistent opioid use remains for hip and knee arthroplasty (Tay et al., 2022). Standard care for orthopedic surgeries includes opioid prescription postoperatively, with approximately 20%–26% of patients at 3 months and approximately 16%–21% at 12 months with sustained opioid use (Tay et al., 2022). Multimodal analgesia in total knee arthroplasty (TKA) has been shown to significantly reduce opioid use, enhance pain relief, facilitate quicker recovery, and shorten the duration of hospital stays though there remains variability in protocols across different studies (Zhang et al., 2025; Qin et al., 2021; Zhao et al., 2024). While prospective protocols demonstrate theoretical potential to eliminate opioids after arthroplasty (Lin et al., 2023), their tightly controlled designs may limit real-world generalizability. Regional anesthesia with peripheral nerve catheters (PNC) has been shown to support a decrease in opioid consumption (Ilfeld, 2011; Ilfeld et al., 2002a; Ilfeld, 2017).

The infusion delivery methods to support these protocols are well established (Finneran and Ilfeld, 2023; Capdevila et al., 2006; DeMaio et al., 2023; Finneran et al., 2021; Ilfeld et al., 2002b; Ilfeld et al., 2004; Ilfeld and Enneking, 2005; Ilfeld et al., 2003b). Infusion pumps used for the delivery of postoperative perineural infusions in the ambulatory and home environments are most commonly elastomeric, with electronic pumps used less frequently (Axley and Horn, 2010). Elastomeric pumps typically deliver fixed rates and may also allow patient-controlled bolus doses, yet electronic pumps can provide automated boluses, delayed start timers, and more precise dosing regimens; electronic pumps have even been shown to enhance the duration and efficiency of analgesia (Finneran et al., 2022; Remerand et al., 2008; Capdevila et al., 2003; Ilfeld et al., 2003b). Additionally, elastomeric pumps often fail to maintain a consistent flow rate: variation in reservoir height, temperature or back pressure (including that introduced by the perineural catheter) can change average flow rates and the delivered infusion volume, whereas this has little effect on electronic pumps (Remerand et al., 2008; Burnett et al., 2011; Hobbs et al., 2019; Capdevila et al., 2003). Use of continuous perineural analgesia in ambulatory surgery, sending patients home with perineural catheters and pumps has increasingly been shown to be effective, feasible and safe in the home environment, when preset programs (Ubanyionwu et al., 2023) and appropriate patient selection, education and follow-up are in place. Notably, 98% of patients have been shown to be comfortable removing their catheter at home (Ilfield e al., 2003a). This case series aims to reflect routine practice conditions, and evaluates the feasibility, safety, and patient-reported outcomes of opioid-sparing analgesia delivery using a smart, connected infusion pump across shoulder, knee, hand and humerus surgeries – no hip or ankle cases in Table 2 supporting perineural blocks and opioid-sparing techniques as part of the Acute Pain Management Program at Georgetown Surgery Center, managed with multimodal OSA protocols and peripheral nerve catheters PNCs.

## Materials and methods

We retrospectively reviewed a small lightweight ambulatory infusion pump (Avoset® Infusion Pump, Eitan Medical) for its technology and applicability in regional anesthesia and postoperative analgesia. Data was collected during an initial pump trial period from August 21 to 8 September 2025, representing the first clinical use of the Avoset pump at this institution. This case series represents a feasibility assessment of implementing smart infusion pump technology for ambulatory perineural analgesia in routine clinical practice. Data was collected retrospectively from patient records from the surgical center, follow-up patient surveys for home PNC infusions conducted over the phone by nurses during their post-operative period from day 3 to day 6, as well as nursing staff interviews. An IRB exemption was obtained from Sterling IRB (Category four, DHHS - ID: 14679-DNelson) on 16 September 2025, confirming that use of retrospective, non-identifiable data did not require prospective ethical approval. All data analyzed were retrospective and non-identifiable.

### Inclusion criteria

Patients were included if they had received their post-operative pain control via the Avoset infusion pump, had undergone orthopedic surgery at the surgical center and received the standard continuous peripheral nerve block (cPNB) protocol. All other infusion pump use and pain control protocols were excluded from the data collection. Data from 20 consecutive orthopedic patients (shoulder, knee, hand, or humerus surgery) managed with multimodal OSA protocols under the Georgetown Surgery Center Acute Pain Management Program was collected.

### Pre-operative analgesia protocol

Peripheral nerve catheters were placed under ultrasound guidance in all patients, with nerve block approach selected according to surgical site: interscalene brachial plexus catheter for shoulder and upper limb procedures, and adductor canal saphenous nerve catheter with or without supplemental iPACK blocks for lower limb procedures. All patients were under general anesthesia with no adjunctive agents were administered to the initial block bolus. All blocks were performed under ultrasound guidance, performed by an attending anesthesiologist, experienced in ultrasound guided blocks. All patients received non-opioid preoperative medicine, including oral analgesics of Acetaminophen: 1,000 mg, Celecoxib: 200 mg, and gabapentin 300 mg. All patients received an initial intraoperative peripheral nerve block with a bolus of 0.5% ropivacaine (20 mL for adductor canal Saphenous catheter and 20 mL for interscalene Brachial plexus catheter or infraclavicular brachial plexus catheter), followed by postoperative continuous infusion via the Avoset pump with 0.2% ropivacaine with patient controlled boluses (a 8 mL/h basal rate with 3 mL patient-controlled bolus, 30-min lockout) with a reservoir volume of 500 mL. Reported risks for catheter displacement have been addressed with enhanced catheter placement with 5 cm insertion as opposed to 10 cm, amongst other solutions (Elsharkawy et al., 2016; Ilfeld et al., 2011). Overall dislodgement rates for perineural catheters in the range of 15%, with rates varying by catheter location (e.g., 5% for interscalene, 25% for femoral) and increasing with time and patient movement (Marhofer et al., 2013; Kalimeris et al., 2020) but the prevalence in the home environment of catheter displacement alone has not been established.

### Post-operative analgesia protocol

Post-operative analgesia consisted of the cPNB infusion as the primary analgesic, with patients being prescribed standardized opioid prescriptions for postoperative pain management, totaling 112.5 morphine milligram equivalents (MME) over a 3-day period. Patients were given 3 options of opioid per 24 h period, over 3 days post operatively. Option 1: Oxycodone 5 mg tablets: Dispense: 15 tablets. Dosing: 5 mg orally every 4–6 h as needed Maximum: 5 tablets per 24-h period. Total MME: 112.5 (oxycodone conversion factor = 1.5) Option 2: Oxycodone/Acetaminophen 5 mg/325 mg tablets (Percocet) Dispense: 15 tablets. Dosing: 1 tablet (5 mg oxycodone/325 mg acetaminophen) orally every 4–6 h as needed. Maximum: 5 tablets per 24-h period (not to exceed 4,000 mg acetaminophen daily). Total MME: 112.5 (oxycodone conversion factor = 1.5). Option 3 (alternative, based on patient preference): Hydromorphone 1.5 mg tablets. Dispense: 15 tablets. Dosing: 1.5 mg orally every 4–6 h as needed. Maximum: 5 tablets per 24-h period. Total MME: 112.5 (hydromorphone conversion factor = 5.0). Opioid prescription was matched with the recommended dosage for Opioid consumption following orthopedic shoulder surgery of 112.5 MME (Vattigunta et al., 2021). All patients will receive concurrent non-opioid analgesics as part of a multimodal pain management protocol: Ibuprofen 600 mg orally every 6 h as needed or naproxen 500 mg orally twice daily as needed (unless contraindicated). Patients were instructed to use opioid medication only when non-opioid analgesics provide inadequate pain relief and to discontinue opioid use when pain is controlled with non-opioid medications alone. Patients were either opioid naive (no opioid use in the 90 days before surgery) or opioid exposed (any opioid use <60 mg morphine equivalent dose within 90 days preoperatively) reflecting real-world populations. No opioid tolerant patients (≥60 mg morphine equivalent dose in the 7 days before surgery) were included.

### Connected data for troubleshooting

The infusion pump data was assessed for any unresolved alarms and effective drug delivery. Infusion data were also captured on a web-based platform reflecting pump data and insights (Eitan Insights®, Eitan Medical®). The Avoset pump transmits data via Bluetooth to the Eitan Insights platform via a designated mobile app, Avoset Go, enabling remote review of pump event data including bolus attempts, alarm logs, and drug delivery. Assessment of this remote monitoring capability will be the subject of a separate dedicated technology evaluation. See Figure 1 for an illustrative example of how the bolus attempts data and alarm data is presented, supporting the hotline for remote troubleshooting of alarms and Figure 2 for an image of the pump connecting to the platform.

**FIGURE 1:**
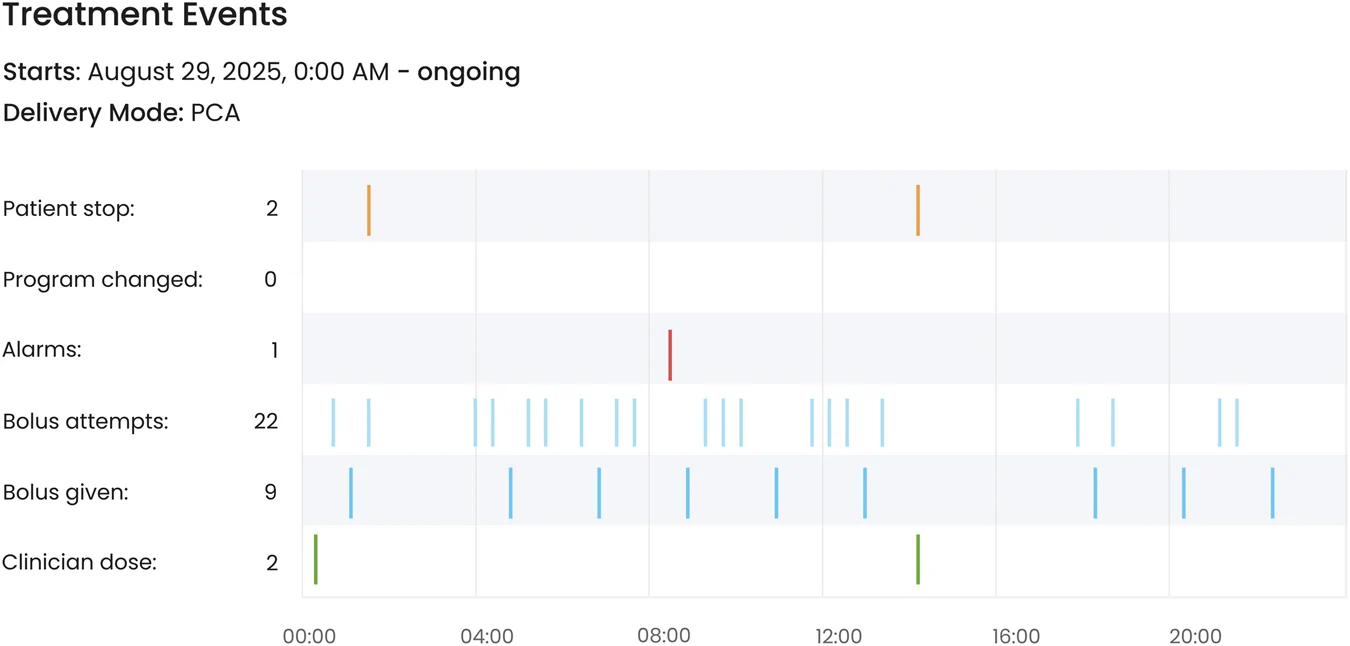
An illustrative example of Eitan Insights dashboard presenting remote infusion data for monitoring.

**FIGURE 2:**
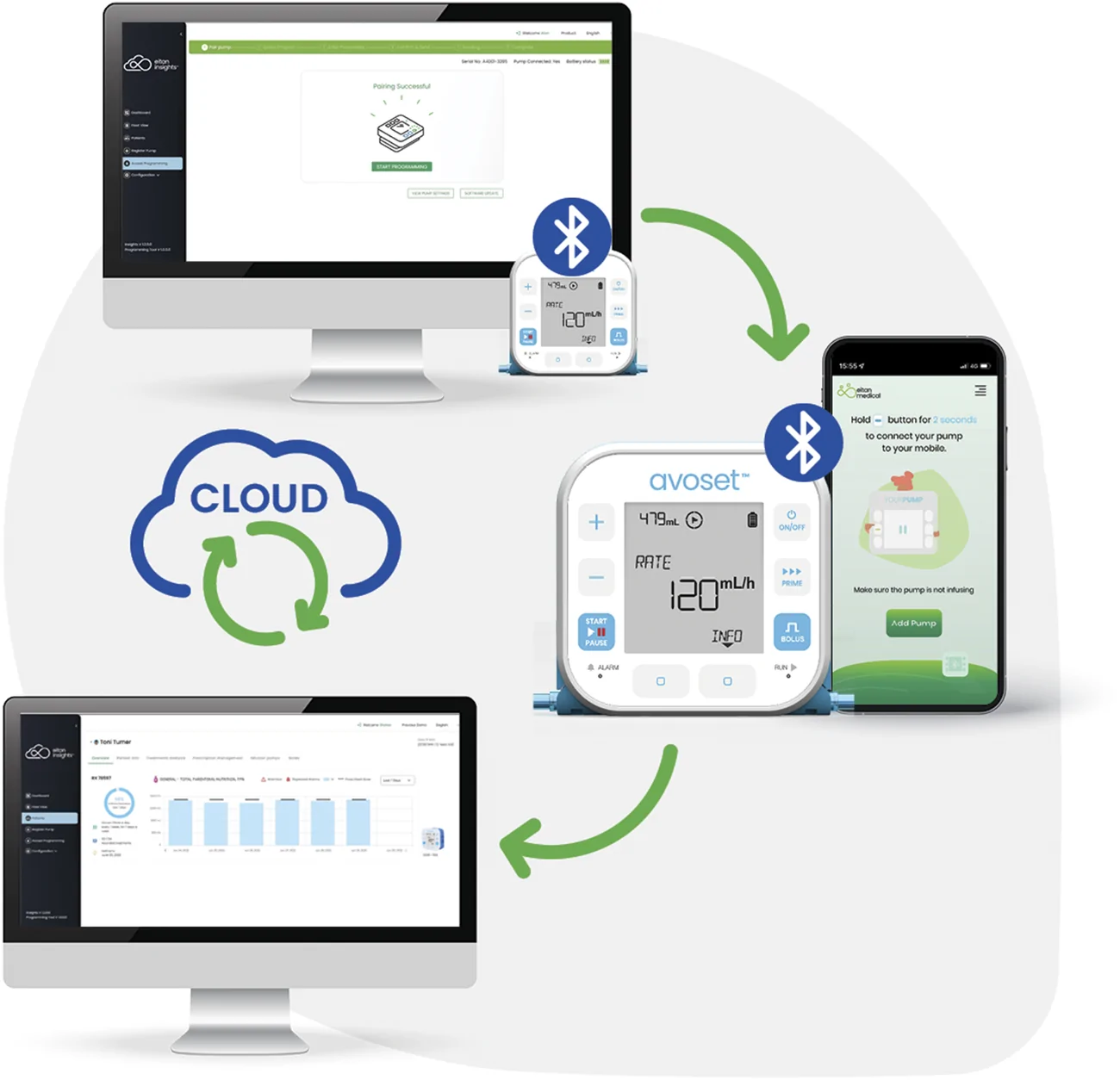
Avoset pump connected via Bluetooth to Eitan Insights platform from the Avoset Go app.

### Post operative data collection

Nursing follow-up was conducted by phone on postoperative day 3–6 using a standardized survey instrument. At each contact, nurses recorded pain controlled or uncontrolled status, PNC intact or removed, white clamp open or clamped, number of opioids taken, opioid name, and any pertinent clinical information. The full survey instrument is presented in Figure 3. Opioid consumption, pump alarms, and infusion completion were recorded to evaluate infusion pump performance, alarm resolution, and successful completion of programmed therapy. Analysis was descriptive in nature, and no inferential statistical testing was performed. Quartiles were calculated by linear interpolation. Proportions are reported with 95% confidence intervals calculated using the Wilson score method.

**FIGURE 3:**
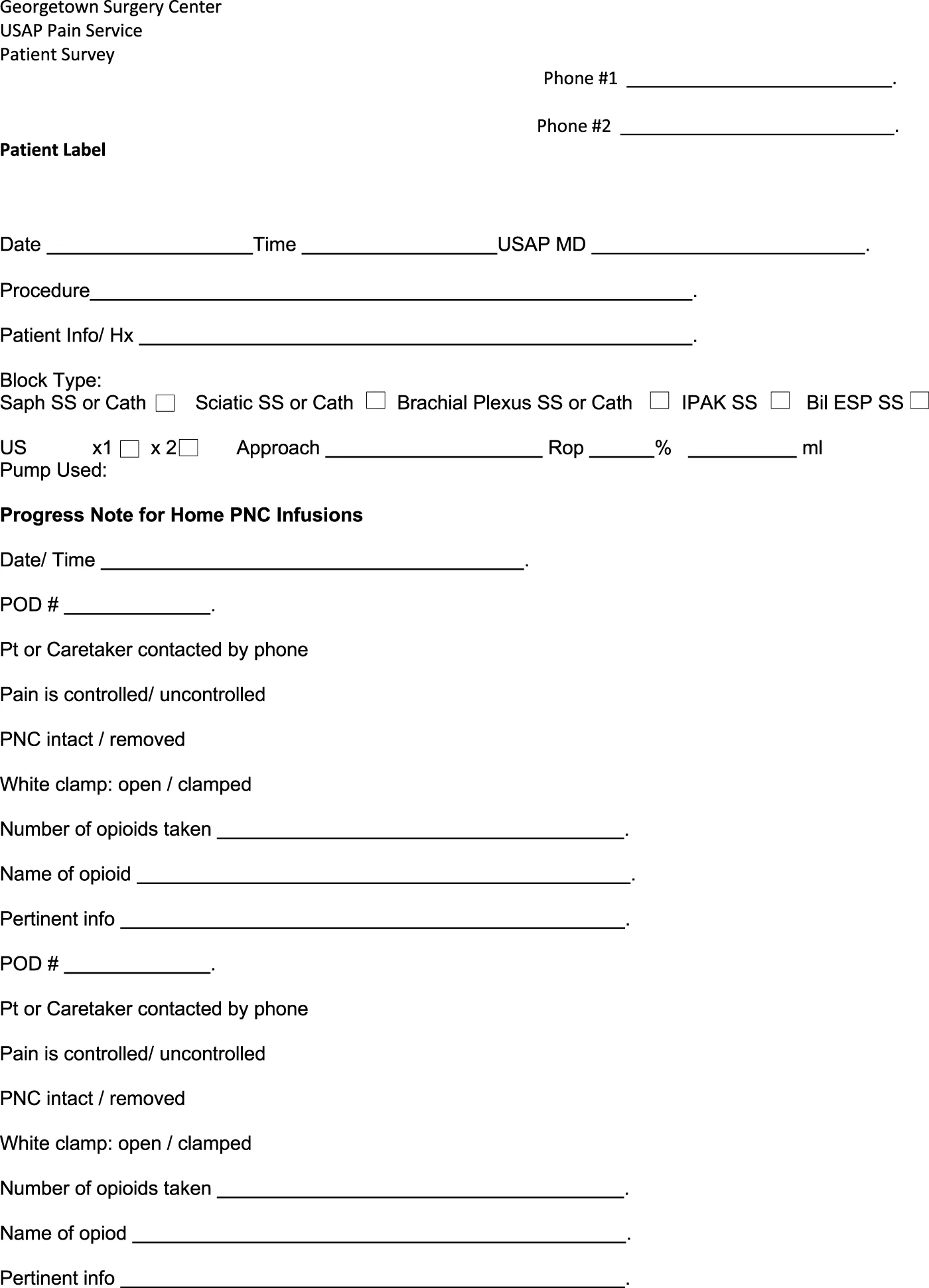
Standardized nursing telephone follow-up survey used for home peripheral nerve catheter (PNC) infusions, Georgetown Surgery Center.

## Results

The survey results show the reaction of patients to uncontrolled pain. The survey found that pain was controlled. This is in the context of the patients’ ability to take opioids in addition to the anesthetic regimen if pain was insufficiently controlled. Baseline patient characteristics are presented in Table 1. Table 2 presents the opioid and dose in the post-surgical period over a period of 3 days, with their infusion outcome.

**TABLE 1:** Baseline patient characteristics.

Patient ID	Age	Sex	ASA classification	Catheter location
1	35	M	ASA I	Infraclavicular brachial plexus catheter
2	28	M	ASA I	Adductor canal catheter with iPACK
3	50	M	ASA I	Interscalene brachial plexus catheter
4	71	M	ASA I	Interscalene brachial plexus catheter
5	64	F	ASA I	Interscalene brachial plexus catheter
6	67	M	ASA II	Adductor canal catheter
7	45	M	ASA I	Adductor canal catheter
8	72	F	ASA I	Interscalene brachial plexus catheter
9	32	M	ASA I	Adductor canal catheter with iPACK
10	23	M	ASA I	Interscalene brachial plexus catheter
11	28	F	ASA I	Interscalene brachial plexus catheter
12	66	F	ASA II	Interscalene brachial plexus catheter
13	55	M	ASA I	Interscalene brachial plexus catheter
14	68	M	ASA I	Interscalene brachial plexus catheter
15	31	F	ASA I	Adductor canal catheter
16	71	M	ASA I	Interscalene brachial plexus catheter
17	63	M	ASA I	Interscalene brachial plexus catheter
18	25	M	ASA II	Adductor canal catheter with iPACK
19	69	M	ASA II	Interscalene brachial plexus catheter
20	61	F	ASA I	Interscalene brachial plexus catheter

All patients received the standard cPNB protocol with 0.2% ropivacaine (8 mL/h basal rate, with a bolus of 3 mL using a 30-min lockout), pre-programmed with the PC tool (Avoset® Programming Tool) and sent by the Avoset Pad via Bluetooth and near-field communication (NFC) technology to the pump. Preset programs are intended to minimize manual entry errors and ensure standardized therapy.

**TABLE 2:** Results of infusion data.

Patient ID	Procedure	Postoperative opioid use over 3 days (MME, drug)*	Pump alarm/event	Outcome
1	R Thumb	None reported	No pump alarms (PNC pulled)	Infusion ended early
2	L ACL	None reported	Air in line alarm prior to treatment start, requiring priming	Infusion continued successfully after priming. Completed infusion as programmed
3	L shoulder	None reported	No pump alarms (PNC dislodged day 2)	Infusion ended early
4	R shoulder	90 MME hydromorphone	No pump alarms	Completed infusion as programmed
5	R shoulder	67.5 MME oxycodone	Reservoir empty	Completed infusion as programmed
6	L TKA	37.5 MME oxycodone	No pump alarms	Completed infusion as programmed
7	L PFL	7.5 MME oxycodone	No pump alarms	Completed infusion as programmed
8	R shoulder	22.5 MME Percocet	Air in line (resolved)	Completed infusion as programmed
9	R ACL	22.5 MME Percocet	No pump alarms	Completed infusion as programmed
10	R shoulder	7.5 MME Percocet	No pump alarms	Completed infusion as programmed
11	L shoulder	None reported	No pump alarms	Completed infusion as programmed
12	R shoulder	None reported	No pump alarms	Completed infusion as programmed
13	L shoulder	None reported	No pump alarms	Completed infusion as programmed
14	R shoulder	37.5 MME Percocet	No pump alarms	Completed infusion as programmed
15	R PFL	45 MME oxycodone	Empty alarm	Completed infusion as programmed
16	R shoulder	30 MME oxycodone	Change bag now alarm	Completed infusion as programmed
17	R shoulder	None reported	No pump alarms	Completed infusion as programmed
18	L ACL	None reported	Occlusion alarm (tubing kink)	Completed infusion as programmed after troubleshooting kink in line
19	R shoulder	90 MME oxycodone	Upstream occlusion alarm	Completed infusion as programmed after troubleshooting
20	L humerus	52.5 MME oxycodone	No pump alarms (PNC dislodged)	Infusion ended early

* All patients were prescribed up to 112.5 MME, over 3 days (15 tablets of either Percocet 5/325 mg or oxycodone 5 mg, or hydromorphone equivalent).

### Opioid consumption

Twelve patients underwent shoulder surgery, three Anterior Cruciate Ligament (ACL) reconstruction, two popliteofibular ligament (PFL), and one each thumb, humerus and total knee arthroplasty (TKA). Pain status was recorded as controlled or uncontrolled at each nursing contact as part of the structured follow-up. Among 20 patients, postoperative opioid consumption was notably low, with a median of 15 MME (IQR: 0–39.4 MME, range: 0–90 MME) over the 3-day postoperative period. Forty percent of patients (8/20) reported no opioid use, while the remaining 60% consumed opioids ranging from 7.5 to 90 MME total. Patients reported their pain as controlled at nursing follow-up. For shoulder procedures (n = 12), median opioid consumption was 15 MME (IQR: 0–45 MME, range: 0–90 MME), with 42% (5/12) requiring no opioids. This is substantially lower than published benchmarks for shoulder surgery with regional anesthesia, where average consumption ranges from 82.5 MME to 112.5 MME, and recommendations suggest prescribing 75–112.5 MME for arthroscopic shoulder procedures (Vattigunta et al., 2021). For ACL reconstruction (n = 3), median consumption was 0 MME (range: 0–22.5 MME), with 67% (2/3) requiring no opioids. This compares favorably to published data showing mean consumption of 28.6–63.8 MME and 46.0–63.6 MME in the first 3 postoperative days with adductor canal blocks (DeMaio et al., 2023). The observed opioid consumption in this cohort was low relative to published reports for comparable procedures (DeMaio et al., 2023). Other upper and lower extremity procedures including TKA, PFL, and humerus fracture (n = 4, 20% of cohort) had a median consumption of 41.25 MME (range: 7.5–52.5 MME) similar to published rates for TKA post operative opioid use (Hendrickx et al., 2025). One thumb surgery patient (5% of cohort) reported no opioid use.

### Pump performance data

Regarding pump performance specifically, no pump-related discontinuations occurred across the full cohort. All alarm events were resolved by patients in the home environment within an hour, without interruption of therapy beyond a short delay while the patient resolved the alarm by troubleshooting the issue. The support hotline was contacted on one occasion only, supporting troubleshooting remotely and all medication was delivered as programmed, with the exception of cases involving removal of the peripheral nerve catheter (PNC). All patients ultimately received the full programmed dose. A total of 7 alarm events occurred Air-in-line (2 events): both required repriming of the administration set. One patient contacted the hotline for guidance; the other independently disconnected the administration set from the PNC, primed, and reconnected. Occlusion (2 events): one occlusion alarm (upstream/downstream not specified) was caused by kinking of the tubing and was resolved by the patient straightening the tubing; one upstream occlusion alarm was caused by the patient lying on the tubing and was resolved by repositioning. New bag needed (1 event): occurred on completion of the programmed volume to be infused and was a post-completion notification requiring no action. Infusion complete (2 events): both occurred on empty bag following completion of the infusion, as expected.

Seventeen of 20 patients (85%; 95% CI 64.0%–94.8%) completed the full programmed infusion. Early discontinuation occurred in 3/20 patients (15%; 95% CI 5.2%–36.0%), attributable entirely to catheter dislodgement or pullout, a known complication of peripheral nerve catheters, and not to any pump malfunction or failure.

## Discussion

In this retrospective case series, ambulatory electronic infusion pump therapy reliably delivered opioid-sparing perineural analgesia in the home environment for orthopedic surgery patients, showing that this infusion pump performed according to specifications. Early discontinuations were attributable entirely to catheter displacement rather than pump malfunction, and all alarm events were resolved by patients without interruption of therapy, alarms were resolved within 1 h and only on one occasion of hotline support in which a nurse supported the patient to troubleshoot with the help of remote data presented on the Insights platform (see Figure 1 illustrative example). All other alarms were resolved independently. These findings support the feasibility of delivering programmed perineural infusions in ambulatory settings using a single infusion pump system and platform that can alert patients to common problems and facilitate troubleshooting at home. No comparative data from other ambulatory pump systems were collected in this study, No comparative data from other ambulatory pump systems were collected in this study, although clinical staff reported favorable experience with troubleshooting and device reliability. 

The IQR of 0–45 MME demonstrates that 75% of shoulder surgery patients consumed ≤45 MME (overall IQR is 0–39.4 MME), which is substantially lower than typical prescribing recommendations of 75–112.5 MME for shoulder arthroscopy and published consumption averages for similar procedures with single-injection regional anesthesia (Vattigunta et al., 2021). This reduction likely reflects the synergistic effect that patient-controlled regional anesthesia has when combined with multimodal analgesia, providing sustained analgesia beyond single-injection nerve blocks (The NO Pain Investigators et al., 2022). It should be noted that the low opioid consumption observed reflects the multimodal OSA protocol as a whole, with the pump playing a key role in supporting reliable delivery of that protocol in the home environment. Prior studies have shown that higher opioid consumption during the postoperative period is independently associated with new persistent opioid use, with each additional tablet increasing the probability of persistent use (Ervin-Sikhondze et al., 2024). While the present study was not designed to evaluate long term opioid outcomes, the low opioid consumption observed is consistent with effective home analgesia during the critical early postoperative period. Other studies have found that most post-surgical orthopedic patients were no longer using opioids at 2 weeks post-operatively (Ervin-Sikhondze et al., 2024). The limited number of additional opioids in this study suggests an alignment with other postoperative opioid-sparing protocols (The NO Pain Investigators et al., 2022; Mandava et al., 2021; Tay et al., 2022).

There has been increasing emphasis on postoperative pain control efficacy, methods and prevention of long-term complications over the last 30 years. US national surveys over two decades (Gan et al., 2014) found the proportion of patients experiencing moderate-severe post-surgical pain remained high (80%–86% in earlier surveys vs. 75% in 2013), yet only 23% of inpatients received analgesia via a patient-controlled device, and only 4% via epidural or nerve block. It is encouraging that patients undergoing painful ambulatory surgery are more commonly receiving cPNB for postoperative analgesia with pump delivery, given that this approach is associated with less pain and reduced opioid requirements in the literature (Ilfield, 2011). Qualitative surveys have shown higher satisfaction associated with PCA use as a pain management option (Ilfeld et al., 2003a).

Electronic pumps support effective infusion, and alarm functionality, while potentially perceived as a nuisance when excessive, ensures that medication is delivered as prescribed. In this cohort, all alarm events were resolved by patients in the home environment without interruption of therapy beyond a short delay that ultimately was resolved and enabled treatment completion. Alarm events including upstream occlusion, new bag, air in line, and priming alarms prompted patients to address common issues such as line kinks, bag replacement, or priming. This highlights a potential advantage of electronic infusion systems over elastomeric pumps, which generally lack alarms and cannot notify users of interrupted flow or occlusion (Hobbs et al., 2019; Capdevila et al., 2003). The ability to review pump event data via the Eitan Insights platform may allow clinicians to analyse treatment data and troubleshoot remotely, capabilities that warrant further evaluation in prospective studies.

The 3 cases of catheter dislodgement or pullout prevented completion of the programmed infusion in those patients. It is important to distinguish these events from pump performance: no discontinuations were attributable to pump malfunction or failure. Although this study was not designed to estimate catheter dislodgement prevalence, the finding aligns with previous reports in the literature. Solutions to address PNC displacement have included ultrasound guidance for catheter placement, proper fixation techniques such as catheter tunnelling, catheter design modifications (catheter-over-needle vs. catheter-through-needle systems), optimal catheter insertion depth, secure dressings and patient education for ambulatory management (Marhofer et al., 2013; Kalimeris et al., 2020). Continuing improvement in peripheral nerve catheter technologies and additional prospective trials could aid accurate catheter placement and simplify perioperative management of patients with peripheral nerve catheters in the home environment (Ilfeld et al., 2011). A qualitative study of perineural infusions at home found that the majority of patients felt safe during their home treatment (Ilfeld and Enneking, 2005). Regular remote check-up from healthcare providers (Agarwal et al., 2021) may be further supported by remote infusion data. Remote pump metrics were not analyzed because remote monitoring data were not a predefined endpoint of this retrospective series. The connected data supported the hotline and remote troubleshooting on one occasion in this study. The assessment of connected data and its contribution in regional anesthesia may be studied in the future.

This study has several limitations inherent to its retrospective case series design. The small sample size and single-centre design limit the generalizability of these findings and preclude robust statistical comparisons. The cohort is procedurally heterogeneous, encompassing shoulder, knee (ACL, PFL and TKA), hand, and humerus surgeries, which introduces variability in postoperative expectations, though all patients received the same standardized cPNB protocol. The absence of a comparator group, such as elastomeric infusion pumps or alternative postoperative analgesic strategies, limits the ability to draw causal conclusions from the observed outcomes. Pain status was recorded only as a binary outcome (controlled or uncontrolled) at each nursing contact, which provides a simple patient-centered indication of pain control but does not capture pain intensity or functional outcomes in the way a validated numerical scale would. Opioid consumption was self-reported via phone survey and is therefore subject to recall bias. In addition, patients were managed within a structured opioid-sparing program, introducing potential selection bias. Although infusion pump event data were available, post-discharge device data were not systematically analyzed, limiting assessment of longer-term performance. Finally, this study was not designed to evaluate long-term opioid use, functional recovery, or cost-effectiveness. Future prospective studies with larger cohorts and comparator arms are warranted to further assess clinical effectiveness and generalizability.

## Conclusion

This study evaluated the feasibility of using a smart electronic infusion pump system to deliver opioid-sparing perineural analgesia in the home and ambulatory environment following orthopedic surgery. The Avoset pump reliably delivered the anesthesia as part of a multimodal OSA protocol across a heterogeneous orthopedic surgical cohort, with no pump-related discontinuations and all alarm events successfully resolved by patients in the home environment with complete delivery of anesthesia according to programming. Opioid consumption was low and consistent with other postoperative opioid-sparing protocols reported in the literature. Pain adequacy was only assessed as a binary outcome to support device feasibility with respect to opioid consumption and device reliability; without a validated scale. Catheter dislodgement rates were consistent with those previously reported, and larger studies are required to ascertain the prevalence of this known peripheral nerve block complication specifically in the home setting. Future prospective studies with larger cohorts and comparator groups are needed to evaluate clinical effectiveness with this system in comparison to elastomeric pumps in the ambulatory and home environments. Additional research should also investigate how remote infusion data and alarm analytics, supported by the Avoset Go App and Eitan Insights platform, can be integrated into follow-up workflows to support postoperative pain management in orthopedic surgery.

## Data Availability

The original contributions presented in the study are included in the article; further inquiries can be directed to the corresponding author.
